# Modelling Cascading Failure in Complex CPSS to Inform Resilient Mission Assurance: An Intelligent Transport System Case Study

**DOI:** 10.3390/e27080793

**Published:** 2025-07-25

**Authors:** Theresa Sobb, Benjamin Turnbull

**Affiliations:** School of Systems and Computing, University of New South Wales at the Australian Defence Force Academy, Campbell, ACT 2612, Australia; benjamin.turnbull@unsw.edu.au

**Keywords:** social phenomena, complex systems, Complex Cyber–Physical–Social System, Cyber–Physical–Social System, Cyber–Physical System, Intelligent Transport System, resilience, Mission Assurance, smart city, critical infrastructure

## Abstract

Intelligent transport systems are revolutionising all aspects of modern life, increasing the efficiency of commerce, modern living, and international travel. Intelligent transport systems are systems of systems comprised of cyber, physical, and social nodes. They represent unique opportunities but also have potential threats to system operation and correctness. The emergent behaviour in Complex Cyber–Physical–Social Systems (C-CPSSs), caused by events such as cyber-attacks and network outages, have the potential to have devastating effects to critical services across society. It is therefore imperative that the risk of cascading failure is minimised through the fortifying of these systems of systems to achieve resilient mission assurance. This work designs and implements a programmatic model to validate the value of cascading failure simulation and analysis, which is then tested against a C-CPSS intelligent transport system scenario. Results from the model and its implementations highlight the value in identifying both critical nodes and percolation of consequences during a cyber failure, in addition to the importance of including social nodes in models for accurate simulation results. Understanding the relationships between cyber, physical, and social nodes is key to understanding systems’ failures that occur because of or that involve cyber systems, in order to achieve cyber and system resilience.

## 1. Introduction

Intelligent transport systems (ITSs) differ from traditional transportation systems in that they include the development of next-generation technologies and interoperate across management, control, and operations frameworks [1]. The integration of wireless technologies, electronic processing, and automation into ITSs enable the development of systems that improve surface transportation safety, efficiency, and convenience when compared with general transportation systems [2]. Intelligent transport systems interlace with an exorbitant number of critical society services, ranging from border coordination, emergency response, public transport routing, personal wayfinding, dynamic logistics, and traffic signal control [3]. These systems further interconnect with other critical infrastructure within smart city grids, such as security microgrids and power grids [4]. ITSs are linked to ventures associated with smart cities, traffic planning, autonomous vehicle control, and vehicle communications [5,6,7]. An outage within an ITS node has the potential to have cascading effects across not only the wider transport system but wider smart city infrastructure, causing devastating outages to critical civil services.

Complex Cyber–Physical–Social Systems (C-CPSSs) are multi-domain systems of systems that exhibit dynamic effects and emergent behaviours. They extend beyond just computational boundaries, interoperating across physical and social nodes within complex ecosystems, with examples spanning from financial markets to electoral events and social media platforms. These systems are interconnected with critical services, and emergent behaviours stemming from the cyber domain can have rippling multi-domain consequences across the wider system. This has been clearly seen in previous events including the Arab Spring uprising [8,9], U.S. Elections [10,11,12], and the CrowdStrike 2024 Outage [13,14]. Such events highlight the need to build cyber systems that are resilient, so that the risk of cascading failure across the wider C-CPSS is minimalised.

Intelligent transport systems are an example of a C-CPSS. They contain cyber, physical, and social nodes interconnected across physical, digital, and human paces, and the nature of the relationships between these nodes can cause complex and emergent behaviours [15].

An individual transport system might be considered a cyber–physical system, but ITS paradigms extend this much further, with significant technology integration across every facet that makes it intelligent. For example, the integration of dynamic routing requires connectivity to weather prediction services, emergency response systems, and other road users to determine optimal routes. Even seemingly simple components of an ITS system, traffic signal control, requires video monitoring, trained machine learning models, and links to emergency response systems. All parts of ITS are dependent on external societal factors and are both impacted by and impact significant social and technical systems in ways that are not immediately apparent.

There is a need to expand the work relating to C-CPSSs, especially as it relates to complex intelligent transport systems. These systems provide critical services to society, and outages at one node in a subsystem can have emergent, unpredictable effects across the wider system of systems. Understanding how these consequences may percolate and how to best mitigate against such emergent disruptive phenomena is of great value to government, emergency services, smart cities, and wider society.

This research presents the following novel outcomes:

- A new synthetic dataset, ODIN-DAWN, that implements specific intelligent transportation system use cases drawing on the ODIN and ARC-IT databases.
- A programmatic model analysis of cascading failure effects, tested with scenarios from ODIN-DAWN.
- A class-level analysis of criticality of the ARC-IT infrastructure.

Understanding and modelling cascading failure and consequences is a specific research need, and this study fulfils this need through the provision of scenario-based case studies that allow for the analysis of failure dynamics. Specifically, this research allows for the examination of cascading failure in order to predict, prevent, and minimise impacts and disruption. Lessons learnt from these simulations can inform cyber-security resilience efforts and enable mission assurance.

The remainder of this paper is structured as follows. Section 2 explains the background of this research. Section 3 describes the methodology used in the design of the programmatic model, and the process applied in each implementation. Section 4 illustrates the results of each implementation, describing key facts and outliers from the model output. Section 5 discusses lessons identified and opportunities for future work relating to this research project and applied C-CPSS studies more broadly. Finally, Section 6 summarises and concludes this paper.

## 2. Background

Intelligent transport systems are smart networks of technological solutions that are applied to traffic and transport, often included within a larger smart-city [16]. ITSs build upon several foundational technologies, including advancements in the Internet of things (IoT), vehicle technologies, the Internet, and the cloud [17]. Due to the interconnectivity and complexity of nodes and dependencies within ITSs, challenges within these ecosystems have the potential to affect vehicles, people, and services [18]. Cascading failure is a significant risk in such networks, as an outage in one element of a network may have service effects that spread beyond the initial point of impact [19,20,21]. A partial or complete failure in one node, used in one process, can impact many others around it, and impact multiple other processes that rely on those nodes, who are in-turn impacting others. Especially within the multi-modal space, there are significant research challenges to mitigate and build resilience against cascading failure [21,22]. ITSs are systems of systems that are complex and lack clear boundaries [23]. Intelligent transportation systems are an active area of research, with specific work on inter-vehicular communication [24], integration into smart cities and other environments [25], and the integration of emerging technologies and paradigms, including Blockchain, smart contracts, and large language models [26,27,28]. Of specific note is the research of Lamssaggad et al., who researched the state of security and resilience across different aspects of ITSs [6]. That work noted both the complex nature of ITSs and that resilience was necessary before mass adoption could be realised. That work noted the need for more research in this field.

This study is framed through the lens of Complex Cyber–Physical–Social Systems (C-CPSSs). These are complex systems that have cyber, physical, and social components that experience emergent phenomena, such as unexpected outages [29,30]. A C-CPSS is a social extension of the cyber–physical system (CPS) paradigm and applies the principles of complexity science to these systems. The literature establishes that intelligent transport systems are complex systems of systems that feature unpredictable emergent behaviour [18,23,31]. Within that context, the artificial systems, computational experiments, and parallel execution (ACP) approach has been developed to enable management plan optimisation, large-scale evacuation, and public traffic scheduling [31,32]. Dhiraj presents a framework for CPSSs in ITS scenarios that highlights research challenges including the lack of formal mathematical models to specify behaviour and the challenge of the feasibility of resilient and robust system design [33]. Connected Automated Vehicles (CAVs) pose specific security, privacy, data analytics, and aggregation issues within CPSSs, especially when integrated within larger system-of-systems architecture [34]. Such systems need to have failure mechanisms with “excellent backup plans” so that vehicle safety is not compromised in the event of an outage, unpredictable behaviour, or adverse weather event [34]. The 4R resilience strategies are also described in the literature, stressing the need to apply smart technologies in cyberspace, optimise physical systems, and enhance social institutions in order to build smart infrastructure resilience [35]. Whilst transportation networks have been considered within the Cyber–Physical–Social System lens [31,36] and the cascading failure lens [37,37], these have never been applied together with the overlay of complexity science to bear lessons for resilience management for mission assurance purposes.

Research into building systemic resilience and reducing the risk of cascading failure in cyber–physical systems extends beyond the ITS case study. Our definition of “resilience” is an extension of Laprie’s, wherein the social component is also considered as part of the capability of complex cyber–physical systems to deliver services that can be trusted when facing change [38]. A C-CPSS that achieves resiliency is able to continuously deliver its intended service outcomes despite any adverse events it experiences [39]. Due to the significant threat posed by cascading failures to network systems, failure modelling and vulnerability analysis studies seek to identify methodologies and defence strategies to increase resilience against disruptive events [40]. Power grids are one area of increased research focus, with strategies such as intelligent approaches for intrusion detection countermeasures, the use of Reinforcement Learning-based Graph Convolutional Networks as a promising solution to cyber-attack detection, and the creation of a three-stage resilience-curve analysis through which mitigation strategies should be implemented [41,42]. Methods such as deterministic and probabilistic simulation, percolation theory, and Markov chain-based models were each evaluated as part of the study, with contemplation of human-in-the-loop factors also highlighted as a requirement for consideration in cascading failure research due to the role that people play in real-time decision-making [41]. Mu et al. propose a cascading failure model where nodal failure consequences can be analysed, with functional load–resilience relationship lessons learnt being applicable specifically to cyber–physical power supply networks [43]. Natural disasters are emergent events that can cause cascading failure in CPSs, and the literature indicates how applying a sequential multi-stage collaborative recovery strategy utilising resilience metrics can significantly improve a system’s ability to absorb shock, maintain services, and recover after disasters [44]. In a manufacturing CPS scenario, models of cascading failure yielded findings regarding the likelihood of physical nodes being the initial cascading failure trigger, where interdependencies were the primary causes of performance degradation in services whilst isolation was the primary cause of performance degradation in physical networks [45].

Although there is active research at the intersection of ITSs and complexity, there are few quantitative data sources for this area. There are several limitations in creating datasets for this field, such as the heterogeneous nature of necessary data, difficulties in data ownership, and privacy concerns. The alternative to this is to generate datasets synthetically. Synthetic and scenario-based datasets have both advantages and limitations. They are often used when it is not practical to collect the depth or breadth of data required in real-world applications, for logistic, legal, or ethical reasons. Such processes are often used in training people and machine learning model validation. However, synthetic datasets are often limited to the algorithm used to generate them, and might not be reflective of real-world situations. Emergent behaviours are also difficult to encode, as they must be at least partially known in advance. To be practically usable, even synthetic datasets must be based on realistic structures and processes. Architectures, case studies, and other models are necessary precursors to creating useful datasets.

One of the global standards in ITS architecture is the United States Department of Transportation’s National ITS Reference Architecture, referred to as the Architecture Reference for Cooperative and Intelligent Transportation (ARC-IT) [3]. This project maintains a national reference architecture and standard in support of ITS deployment and collaborates with partners internationally [46]. ARC-IT is comprised of the key types of nodes, relationships, and workflows for how they interact across different tasks. The ARC-IT architecture has multiple connected “views” that depict different aspects of an ITS. These include physical, communications, functional, and enterprise views. The same object might be present across multiple views, with different properties in each. The architecture has been applied to connected autonomous vehicle research to gain a deeper understanding of business opportunities, investment opportunities, and challenges associated with the autonomous vehicle ecosystem [47]. The architecture has been aligned to digital forensics readiness best practices and has informed the identification of key ITS technologies such as such as electronic toll collection [48,49]. ARC-IT has also been used in the development of user needs’ frameworks based on activity theory, which has further applications to next-generation autonomous ITSs [50].

The OE Data Integration Network’s (ODIN) Decisive Action Training Environment (DATE) is another significant data source that may be applicable to ITSs and other synthetic datasets. Designed for military training, DATE is one of the largest fictional unclassified scenario data repositories publicly available [51]. DATE is comprised of high-fidelity manufactured scenarios, applicable for strategic, operational, and tactical data. Scenario information is varied and includes countries, cities, geographic maps, political parties, and notable individuals. This environment was originally designed to provide relevant common training scenarios based on open-source information [52]. Outside of official military exercise applications, DATE has been used to train AI-enabled adversaries as part of immersive simulation environments [53]. Research reports have further been generated from ODIN as inputs into larger wargame datasets including the Information Warfighter Exercise Wargame [54]. Due to its open-source nature and public release, the datasets are well positioned for corporate, military, and research access purposes.

ARC-IT and DATE fulfil different but complementary functions. ARC-IT describes generic workflows and processes but not exactly how they would be implemented. For example, ride-sharing for shared-use mobility is discussed but not specific to one or more companies. All processes and views are conceptual. By contrast, DATE does not consider ITSs at all but provides a rich landscape of synthetic instances that can be used for a wide variety of purposes. DATE is also useful in that all data derived are clearly not based on real data.

Ultimately, this research sits at the intersection of intelligent transport systems, C-CPSSs, and dataset creation to apply methods for modelling cascading failure. This is in support of understanding and increasing the resilience of complex social systems. The literature has informed us how ITSs and C-CPSS have been modelled in this capacity, but there is a clear lack of data to allow quantitative approaches in this field. Whilst the literature does contain some scenario and digital-twin models, these are limited in scope, often to power or manufacturing networks. As noted, it is difficult to ensure data are broad and complex enough to understand cascading failure.

This work seeks to extend current work in modelling complexity in ITSs by applying use cases from ARC-IT and combining them with the scenario data from ODIN to create valuable, realistic cascading failure models. With such data, it is then possible to realistically model cascading failures across connected systems. This work seeks to develop a greater understanding of cascading failure in ITS implementations, but to do this, the data must first exist to evaluate them.

## 3. Methodology

This research was comprised of two parts: first, the development of a dataset capable of modelling the workflows used in ITS implementations, and second, to develop a programmatic model to examine the cascading failure effects within C-CPSSs. The dataset was then used to evaluate the programmatic model. This new dataset was named ODIN-DAWN.

As noted, ARC-IT is a global standard in ITS architecture developed by the United Stated Department of Transportation and is used to enable the implementation of intelligent, integrated, collaborative transportation systems [3,46]. ARC-IT was selected as the structural backbone for the ITS system design for this project due to the comprehensiveness of the standard, with hundreds of system nodes, relationships, and service packages encompassed within the architecture. This built a reliable, validated foundation of a C-CPSS service design onto which scenario data could be build and cascading failure tests could be implemented.

To create the new dataset, ODIN-DAWN, three service packages from ARC-IT were chosen to represent the system-of-systems ecosystem. These were the Hazmat Management, Carrier Operations and Fleet Management, and Emergency Response packages. Contextual information from the ODIN’s databases was then used to generate an implementation of each of those use cases into the scenario environment, particularly relating to the DATE fictional state of Khorathidin. For the generation of fictitious information, such as number plates and names, a large language model was used. An excerpt of the nature of the information generated as part of ODIN-DAWN can be found in Table 1.

The items in this table are illustrative of different types of exemplary ITS nodes that can be represented within the C-CPSS model. Each node has the potential to exist within the cyber, physical, and/or social domains concurrently. A different input file is then used to represent the complex relationships between each node. In total, the model expects five files as inputs to build the C-CPSS. These contain node information, as in the above table, node security data, relationship information, relationship security data, and relationship communications data.

The programmatic model used for this project was developed with the intent to accurately simulate all systems and subsystems within a C-CPSS and then annotate the interdependencies between the systems so that the effects of cascading failure could be measured. To develop the programmatic model, the Python programming language was used, with the networkx and matplotlib libraries. It was designed to be compatible with the exported data structures in the ARC-IT architecture and using scenario data from the ODIN databases. There were two implementations of the programmatic model. The first implementation was a full export of all service packages from the ARC-IT, which was only implemented at the class level. This meant that the systems and their relationships were represented without a scenario implementation. This implementation was referred to as the ARC-IT class implementation. The second implementation involved the ODIN-DAWN scenario development, referred to as the ODIN-DAWN functional use case. This process is outlined above. The first model provided a high-level overview of all classes, whereas the second provided detail for the implementation of specific use cases and their interaction, allowing for an exploration of ODIN-DAWN.

Input data for the model were exported from a subset of the ARC-IT databases, parsed via a series of CSV files, and augmented within the program. These files contained details such as node names, relationships, and types. Once input data were parsed, the program was able to calculate the functions as per Table 2.

The program takes the provided inputs and builds the C-CPSS virtually, inclusive of the nodes and relationships. Nodes can be cyber, physical, and/or social, and exist at the ARC-IT architecture layers of enterprise, physical, functional, or communications. The system can then be queried to determine key insights regarding its criticality, key nodes, and robustness. The difference in effect between a traditional cyber–physical system model and the C-CPSS model in terms of calculating the percolation of cascading failure consequences was a key output of the model that served to justify the C-CPSS label.

## 4. Results

As noted, two models were developed. The first operated on the ARC-IT class-level detail and was called the ARC-IT class implementation. The second programmatic model used the novel ODIN-DAWN dataset. The results from these programmatic models are discussed separately, as they measure different aspects of the work.

### 4.1. ARC-IT Class Implementation

The first programmatic implementation involved incorporating the entire ARC-IT database. This implementation involved data that served as the blueprint architecture behind the ARC-IT system but did not involve a use-case example as in the ODIN-DAWN implementation. There were 134 communications profiles within the dataset, 985 information flows, and 583 individual system nodes in the ARC-IT implementation. Each type of information flow had multiple sources and destinations. Systems and their interdependencies with each other at the physical, functional, enterprise, and communications layers were graphed using the model.

The entire graph was not completely connected, and there were some nodes that did not link with any other nodes. This is representative of the current ARC-IT architecture. Based on this, no node could have 100% graph reachability due to these outlying disconnected systems. The impact of this is discussed in the Analysis Section.

**Figure 1:**
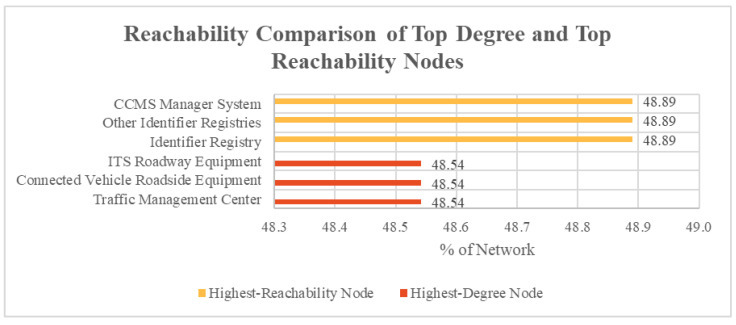
Reachability comparison of top degree and top reachability nodes.

Across the network, the cyber nodes that contained the highest degree, that is, the largest number of outer connections are illustrated in Figure 1. With its 150 outward connections, this meant that the Traffic Management Center was directly interconnected with 25.7% of the total network and indirectly connected to 48.54% of the wider network, highlighting how central it was to the overall system. Interestingly, Figure 1 also shows how the nodes with the highest degree differed from the nodes with the highest overall reachability. Of all the nodes within ARC-IT, three returned a shared equal-first highest reachability across the network. With them as a starting point, these nodes were able to reach 48.9% of the wider network, meaning a failure at one of these could cause outages to almost half of systems listed. Of these highest reachability nodes, all had a degree of five or less. This reveals that degree is not necessarily an indicator of total nodal criticality, but high degrees can correlate with high reachability.

**Figure 2:**
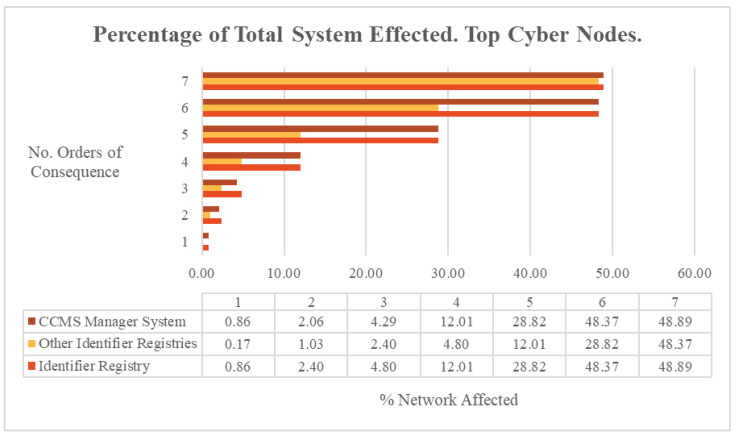
ARC-IT percentage of total system affected. Top cyber nodes.

The cascading consequences of an outage at these critical nodes, as defined by reachability, diffused at different rates for each system, as shown in Figure 2. Whilst they all eventually achieved the same network consequence percolation, the Identifier Registry was the node that achieved the widest distribution by the second order of consequence and maintained this for the third order of consequence. The implications for this are discussed in the Analysis Section.

One metric used to determine the value of the simulated C-CPSS was to compare the fidelity of cascading failure results in a simulation that only considered cyber and physical nodes, versus a simulation that considered cyber, physical, and social nodes. The Identifier Registry and Other Identifier Registries were so reliant on the inclusion on social nodes for consequence diffusion that their reachability across the graph changed by 48.7%. Similarly, the reachability for the CCMS Manager System was also highly reliant on social nodes for consequence diffusion, with its reachability changing by 48.5%.

**Figure 3:**
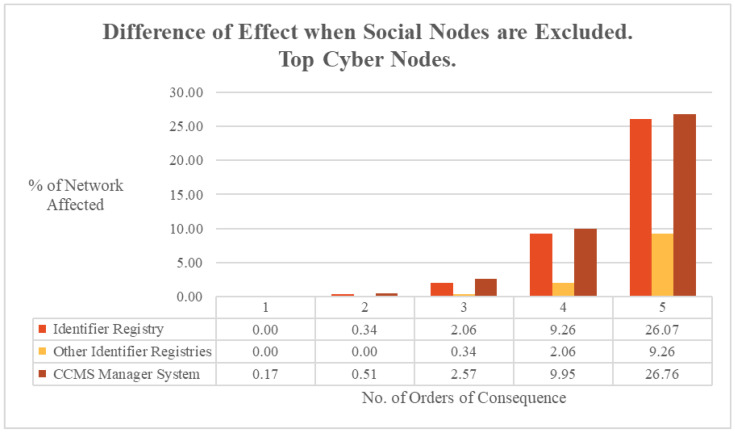
ARC-IT difference in effect when social nodes are excluded. Top cyber nodes.

The difference that exclusion of social nodes made to cascading consequences’ calculations was also considered for each order of consequence. As shown in Figure 3, the difference in consequence percolation started at zero or a minimal value, but by the fifth order of consequence, it had varied between 9 and 26.8% of the network.

The communications profiles used to enable the systems within the ARC-IT architecture were also examined as part of this implementation. The communications profiles that were relied upon most heavily for system links and communications were smart-city communication protocols, including DATEX, NTCIP, and OMG DDS, and Secure Internet protocols. This indicates that they are the most significant and prolific underlying communications infrastructure for ITSs. As a consequence, they act as the communications backbone to the system, where a potential vulnerability, disruption, or outage could cause significant cascading consequences to the reliant information flows.

The top four most critical communications profiles, defined by their highest connectivity reliance, were analysed regarding their cascading failure consequences to the wider system of systems. Whilst additional orders of consequence did increase the portion of the network affected by 1–5% overall, this was a minor amount in the context of the first order of consequence impacting 44–49% of the network immediately. These statistics indicate that communications profiles are pervasive enablers within the system.

**Figure 4:**
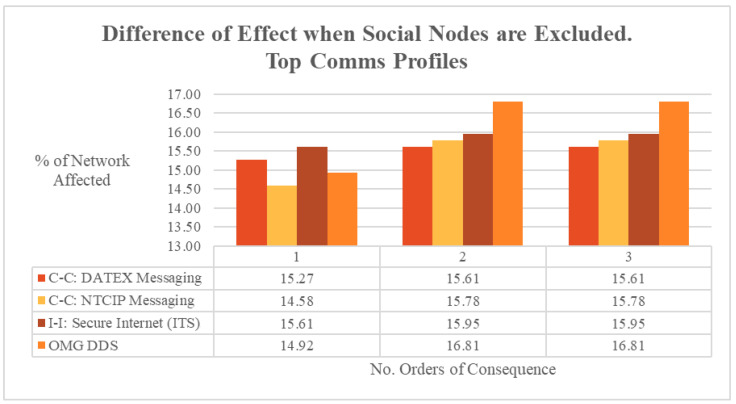
ARC-IT difference in effect when social nodes are excluded. Top Communications Profiles.

The impact of excluding social nodes in determining the impact of a communications profile outage was also calculated for the most critical profiles. This difference is shown in Figure 4, where the exclusion of social nodes reduces the accurate simulation of consequences across the wider graph by 14.6–16.8% overall.

### 4.2. ODIN-DAWN Functional Use-Case Implementation

The implementation of the ODIN scenario data into the programmatic model aligned with the blueprint structures designed in the ARC-IT database. The ODIN-DAWN implementation contained three service packages. There were 33 implemented communications profiles, 37 information flows, and 465 system nodes. Each type of information flow had multiple sources and destinations.

Graphically, the system of systems organised into three main clusters that roughly corresponded to each of the three use cases applied to the dataset. Thematically, these were Hazmat Management, Carrier Operations and Fleet Management, and Emergency Response. Whilst east cluster was locally defined, all clusters were interconnected into each other as part of the larger network to create the system of systems.

**Figure 5:**
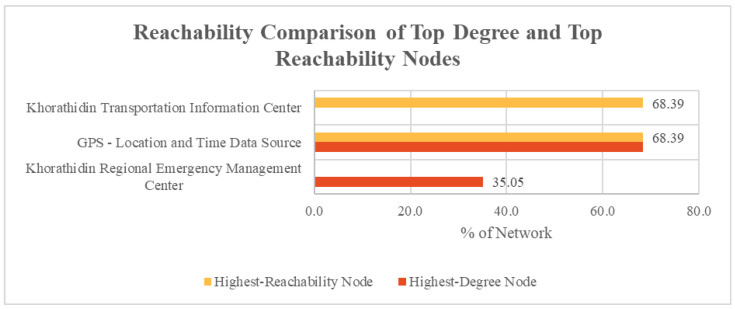
Reachability comparison of top degree and top reachability nodes.

Across the network, the cyber nodes that contained the highest degree and the highest reachability are compared in Figure 5. There was a moderate correlation between degree and reachability in that implementation, with the GPS—Location and Time Data Source node being both the node with the highest degree and the highest reachability. However, the co-highest top reachability node, Khorathidin Transportation Information Center, did not correlate with being in one of the top cyber nodes with highest degree values.

**Figure 6:**
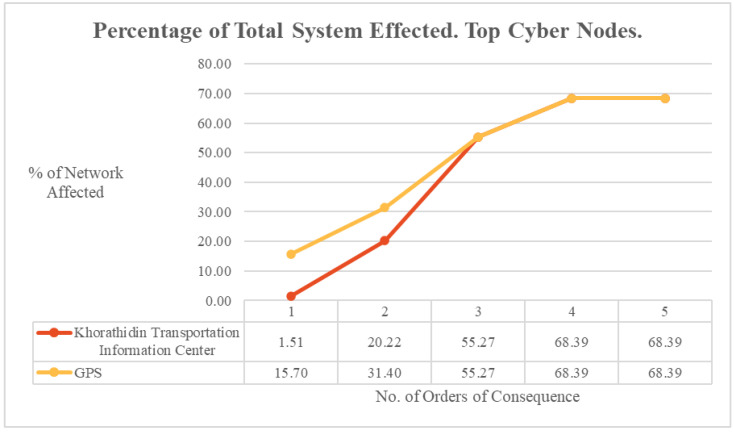
ODIN-DAWN percentage of total system affected. Top cyber nodes.

The cascading consequences of an outage at the two most critical nodes, as defined by reachability, percolated at differing rates across the wider system, as shown in Figure 6. By the third order of consequence, both nodes reached equivalent diffusion rates that remained consistent for the remainder of the consequence diffusion. However, during the initial first two orders of consequence, the impact of a system outage against the GPS node affected a larger percentage of the overall system than an outage against the Khorathidin Transportation Information Center. The consequences of these figures are discussed in the Analysis Section.

Again, in this implementation the value of simulating C-CPSS was determined through comparing the fidelity of cascading failure results in a simulation that only considered cyber and physical nodes, versus a simulation that considered cyber, physical, and social components. Nodes in the system were highly reliant on the inclusion of social nodes in the simulation in order to accurately simulate the effects of cascading failure. The biggest difference in graph diffusion when social nodes were excluded was for the Khorathidin Transportation Information Center and the GPS nodes, which both experienced a reachability change of 68.2%. Additionally, other nodes that were significantly affected included the Bassac Traders Inc. Commercial Vehicle Administration Center and the Khorathidin Petroleum Commercial Vehicle Administration Center nodes, which through exclusion of social nodes experienced a reduction in consequence reachability of 48.0%. These statistics highlight the widespread impact that the inclusion of social nodes makes to accurate consequence simulation.

**Figure 7:**
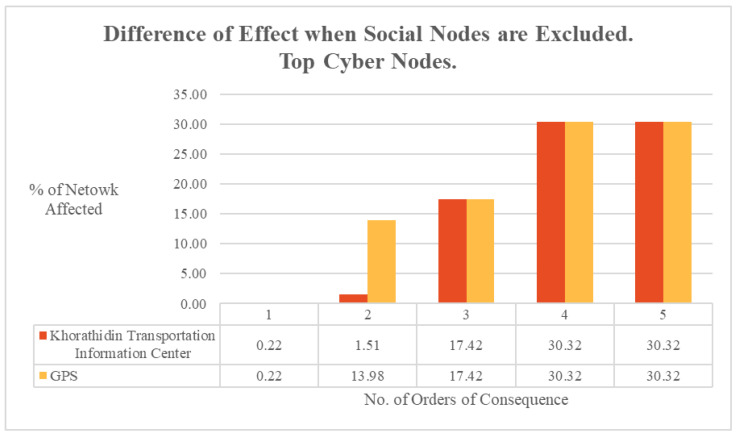
ODIN-DAWN difference in effect when social nodes are excluded. Top cyber nodes.

The difference that exclusion of social nodes made to cascading consequence calculations was also considered by each order of consequence for the top cyber reachability nodes. As illustrated in Figure 7, the second order of consequence caused a significant difference in impact of the effect between the Khorathidin Transportation Information Center and GPS nodes, with it having a relatively limited impact on the former at 1.5% of the wider system but affecting the latter nearly ten times as much at 14.0%. However, this diffusion difference had dissipated by the third order of consequence, bringing both statistics into an equivalent rate of impact for the remaining orders of consequence.

The communications profiles used to enable the systems within ODIN-DAWN were also examined as part of this implementation. With an increase of nearly 1.5 times the number of system links as its closest competitor, the top communications profile in the ODIN-DAWN implementation was the simple wireless network protocol SNMPv3/TLS. Other profiles, including wireless internet protocols and smart-city enabling protocols such as NTCIP, were also heavily utilised, however not to such an extensive extent. Enabling technology such as general internet protocols, having freedom and disruption-free wireless communications and available encryption mechanisms for cyber security all have the ability to influence the implementation of this communications profile within the ITS. Because network protocols were so prolific throughout the system, they also posed as a potential single point of failure. A cyber-attack, new engineering vulnerability, or software bug had the potential to completely disrupt the data flows that relied on each profile, having flow-on disruptive effects to wider ITS services.

The top four most critical communications profiles, as defined by their highest connectivity reliance, were analysed regarding the cascading consequences of a nodal failure to the wider system. The most critical communications profiles were able to achieve a 68.2% network impact saturation by the second order of consequence before stabilising at this rate. For the top three profiles, more than half (55%) of the network was permeated with consequences as a direct result of their outage. For example, when the wireless simple network protocol experienced a service outage, the first-order consequences affected a range of services, from Commercial Vehicle on-board equipment to Chao Phraya Imports Commercial Vehicle Administration Center to fleet freight managers, and those services provided by the Pnom Pehn Emergency Management Center. Such an outage would be so significant the majority of information flow services would be potentially directly degraded.

**Figure 8:**
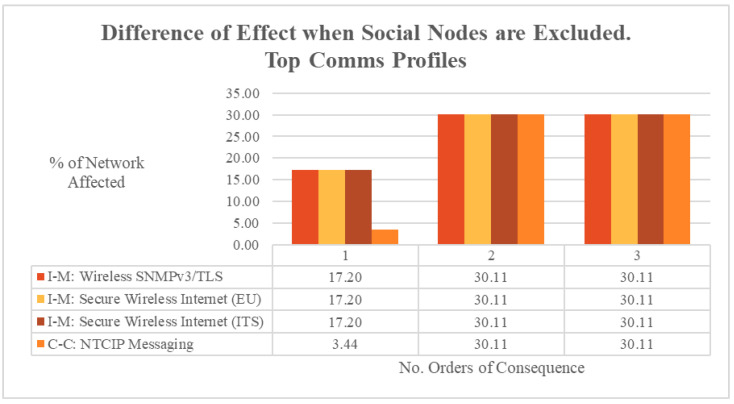
ODIN-DAWN difference in effect when social nodes are excluded. Top communication profiles.

The effects of excluding social nodes when determining the consequence of a communications profile outage were also calculated for the most critical profiles. The difference in calculated network impact is shown in Figure 8. When each communications profile fell, by the second order of consequence, the simulation’s calculated impact to the network was 30% less when social nodes were excluded. This meant that traditional models that only considered the cyber–physical aspects of an outage would miscalculate the consequences of an outage by up to 30% because social nodes and their interactions were not considered. This highlights the significance of the social dimension in modelling C-CPSSs for ITSs, and its role in linking nodes of all domains together.

## 5. Analysis

The two implementations of the programmatic model, through the ARC-IT class implementation and the ODIN-DAWN functional use case revealed lessons and opportunities for C-CPSS research. A summary of these lessons learnt include the limitations of applying graph theory, how orders of consequence inform rate of impact, the importance of including social nodes, the decisive nature of communications profile outages, the need for systemic prioritisation and fluidity, and the need for a mission assurance overlay. Finally, an opportunity is identified and recommended to integrate the programmatic model into a tabletop or simulated exercise to enhance its value to critical service sectors.

### 5.1. Limitations of Graph Theory

Relying on graph theory calculations were limited by computational complexity and therefore processing resources’ availability. For both implementations, calculations such as reachability and degree were relied upon as inputs into decisions and functions within the code. Increasing the number of nodes within the graph, that is, the number of Cyber–Physical–Social Systems and the number of interlinks between them, increases the overall computational complexity of the graph. Such computational complexity requires significant processing resources and time to calculate.

The impacts of this resource requirement are seen in the computation of the communications profiles’ cascading consequences, where only three orders of consequence were calculated compared to cyber-node cascading consequences which were calculated to five orders of consequence. The degree of the most critical communications profiles in the first implementation was over 19,000 links, compared to a degree of the most critical cyber node being up to 5 links. Comparatively, calculations for the first order of consequence are much more computationally expensive for the communications profile compared to the cyber node, with this trend continuing for each additional order of consequence. Subsequently, for the ARC-IT implementation, consequences for cyber nodes were calculated to the seventh order of consequence whilst consequences for communications profiles were calculated to the third order of consequence. For the ODIN-DAWN implementation, consequences for cyber nodes were calculated to the fifth order of consequence whilst consequences for communications profiles were calculated to the third order of consequence.

Graph theory is a strong basis for modelling C-CPSSs that can be used to provide some initial insight into node analysis for criticality. However, its methods are too computationally expensive for it to be a complete solution for C-CPSS simulation for cascading failure simulation and critical node identification, as true C-CPSSs have the potential to be significantly larger and more complex than the datasets tested within the implementation environment. Future work should still incorporate less computationally demanding metrics of graph theory, such as degree, into their models in order to assist in the evaluation of nodal importance.

Disconnected regions of the system-of-systems graph were included in nodal reachability statistics, potentially skewing the perception of impact percolation across connected systems. The ARC-IT class implementation in particular had a set of system nodes that were not connected to other nodes within the C-CPSS, but those nodes were still counted as part of the overall reachability total for statistical purposes. As there was no link to those lone nodes, no other node within the system could ever theoretically achieve a reachability of 100%. The ODIN-DAWN implementation experienced this phenomenon significantly less. Future work should include the option to hide lone disconnected systems from the network map, so that more accurate reachability statistics can be calculated between all feasible nodes and their connections.

### 5.2. Order of Consequence Informs Rate of Impact

The percentage of the overall graph that a node could reach was used to assist in determining its criticality to the system, but the number of orders of consequence taken to achieve that statistic is also important in critical-node cascading-failure management.

**Figure 9:**
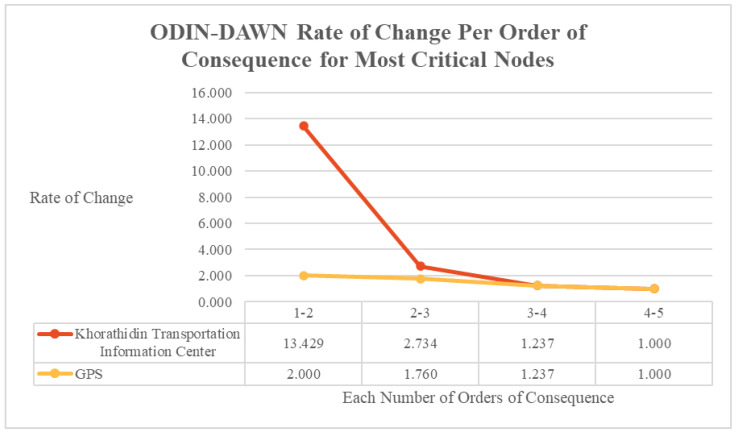
ODIN-DAWN rate of change per order of consequence for most critical nodes.

This is illustrated in the ODIN-DAWN implementation, where both the Khorathidin Transportation Information Center and the GPS nodes, by the fourth order of consequence, permeated outage impacts to 68.4% of the entire network. However, each node percolated outage effects at a different rate, with the GPS node having greater than ten times more initial impact (15.7%) to the whole network on the first hop than the Khorathidin Transportation Information Center (1.5%). In that same case of the ODIN-DAWN implementation, the steepest rate of percolation change occurred between the first and second order of consequence for the Khorathidin Transportation Information Centre, as highlighted in Figure 9. This analysis provides three critical pieces of information to support cascading-failure management decisions. The two nodes had the same overall total reachability; however, the GPS node had the greatest immediate impact to the system and the Khorathidin Transportation Information Center had a significant second-order impact to the system. Breaking down the model and considering more metrics than just reachability provides deeper insight into how a cascading failure event could potentially occur, and how that event would spread throughout the network. When making decisions regarding which nodes to prioritise in efforts such as cyber-security uplift or hardening, all of these data need to be considered in order to inform management.

**Figure 10:**
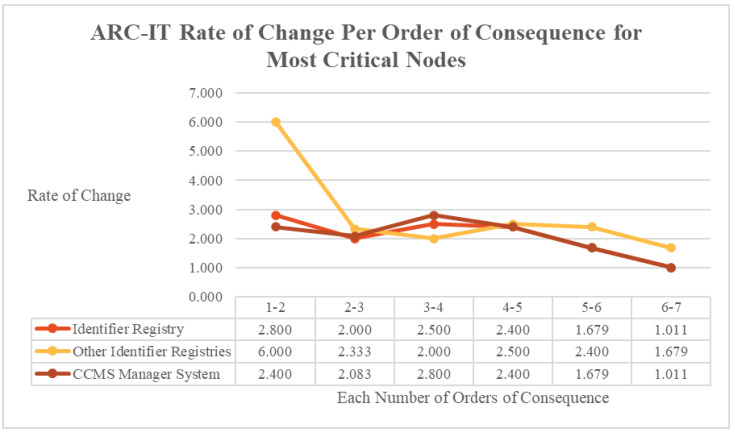
ARC-IT rate of change per order of consequence for most critical nodes.

Considering the percolation rate per order of consequence also reveals lessons in applying nonlinear dynamics. In the ARC-IT implementation, the rate of change per order of consequence for the most critical nodes did not follow a linear or formula-driven path, as shown in Figure 10. Nonlinearity in the field of complexity refers to the idea that small fluctuations can cause significant repercussions and that systems are responsive to such fluctuations [55]. In a mathematical sense, it refers to systems in which the superposition principle does not apply [56]. Whilst all three nodes may have the same overall reachability, they internally do not conform to consistent rates of change or percolation. Just because two nodes have the overall same cascading failure reachability does not make them fungible in terms of their other qualities worth considering in the context of criticality. By this measure, applying the Complexity label to CPSSs is justified, as nonlinearity is a key component of complex and dynamic adaptive systems.

There are benefits to decision-makers in knowing the rate and spread of consequences at each order of effect in the event of an outage. This data is especially enhanced when information regarding service delay can be incorporated, such as the expected time to transition from first-order effects to second-order effects.

Some nodes may be immediately impacted by an outage, whereas others may be less immediately vulnerable and only degrade over time. An isolated example of this would be the loss of an Active Directory server on a Local Area Network. Users on Windows computers would still be able to log into their accounts if they had credentials cached on those hosts, but this would only delay the inevitable service outage stopping logons across the entire network. Conversely, an outage at the main router connecting the Local Area Network to the Internet would cause an immediate Internet-access service degradation without delay. When prioritising cascading-failure management and business continuity efforts, it is valuable for cyber-security experts to consider these impacts at each order of consequence so that they are able to prioritise efforts based on organisational needs and risk tolerances.

### 5.3. Importance of Including Social Nodes

The impact of including social nodes in C-CPSS simulations was significant to cascading-failure calculations in all implementations. Whilst the most critical cyber nodes, as defined by total network reachability, were not also categorised as social nodes, social nodes still played a significant role in the distribution of cascading-failure effects across the wider network. Social nodes had the ability to essentially interlink “air-gapped” systems, passing on consequences that otherwise would not have been captured.

The value of the social component of these systems cannot be underestimated, with up to 30% of consequences not captured on graphs that did not include social nodes. For cascading failure to be accurately modelled, all components within a system need to be accurately represented, and this requires the identification of the social elements within C-CPSSs.

### 5.4. Communications Profile Outages Are Decisive

In both implementations, an outage of a key communications profile had a decisive impact on the system almost immediately. Traditionally, military units were considered ineffective when they reached a casualty rate of 40% [57]. The ODIN-DAWN dataset was built to meet U.S. Army training scenario needs. Based on the simulation, the first-order consequence of an outage of any of the top four communications profiles would immediately degrade between 41 and 55% of the entire network. Whilst the examples in ODIN-DAWN are based around civilian infrastructure naming conventions, the use cases chosen align specifically with cases that have military cross applications.

If an Army unit was in a scenario that aligned with the use cases simulated in ODIN-DAWN, such as Emergency Response, Carrier Operations and Fleet Management, and Hazmat Management, it is feasible that a single communications profile outage could wipe out up to 55% of the network immediately, cascading into service degradation of up to 68% over time. From a military capability perspective, this is a significant and potentially decisive outage that could have devastating impacts to operations and missions.

Homogeneity and lack of diversification in alternative communications profiles only increase this risk. These implementations highlight how cyber elements are critical to wider C-CPSS and organisational functions. The loss of a cyber service can have dramatic permeating effects to the wider system beyond the cyber domain, and thus building resilience in that system against outages is imperative.

### 5.5. Need for Systemic Prioritisation and Fluidity

Future model iterations should be more adaptive to changing graph structures and criticality fluctuations over time to more accurately represent real world C-CPSSs. Sensitivity to initial conditions and feedback loops are two features of complex systems which can cause deviance and nonlinear outcomes [58]. In the version of the programmatic model used, there was no opportunity to modify graphs or structures over time, limiting the ability of the simulation to represent these aspects of complex phenomena. This would have been especially valuable in the ODIN-DAWN implementation, as certain environmental events may have changed the criticality or network structure of the graph over time. For example, for the Carrier Operations use case, a truck going offline whilst in maintenance would affect the allocation of a commercial vehicle driver to that truck. Additionally, if a weather event such as a wildfire occurred, it is likely that the criticality of nodes associated with the Emergency Response use case would increase over the other use cases temporarily. Developing a C-CPSS model that captures and allows for this systemic adaptivity would therefore provide greater accuracy to these simulations, allowing researchers to more exactly identify critical nodes and subsystems at risk of cascading failure.

### 5.6. Need for Mission Assurance Overlay

Future work should venture to apply a mission assurance analysis overlay on top of the network graph in order to represent temporal organisational priorities. The ARC-IT architecture attempts to separate nodes into “views” which define the scope of relationships, being communications, physical, functional, and enterprise. This mechanism assists in assigning meaning and purpose to CPSS nodes but does not inherently convey the requisite data needed for informed mission assurance decision-making.

Definitions of criticality need to be defined but multi-faceted. In the implementations, the criticality of a system node was calculated based on its reachability to the rest of the network, and for communications profiles, it was based on the number of connective links relying on that profile. This is a one-dimensional approach to determining criticality that does not take into account the myriad of other potential factors that could contribute to the criticality of a node within a network at any given time.

Without the context of mission assurance information, a C-CPSS exists in the vacuum of hardware, software, and data in which it was created. The importance of nodes can therefore only be evaluated within the context of the network itself. In a mission assurance context, systems exist to serve a purpose beyond their architecture to a wider organisational goal. Therefore, overlaying this mission and translating its required services into priorities in the C-CPSS domain is required in order to accurately identify critical nodes within the C-CPSS.

Building such a mission assurance analysis overlay and integrating it with a C-CPSS cascading-failure simulation model is an opportunity for future research. The outputs of this project would be extremely useful in building resilience in critical service and architecture, therefore reducing the risk of widespread catastrophic failure in the event of an outage or attack.

## 6. Conclusions and Future Work

Intelligent transport systems are a maturing service architecture that interlink with pivotal societal services within smart cities including emergency management, critical infrastructure, and power grids. Building resilience within ITSs is essential, because they are inherently vulnerable to emergent disruptive events. As Complex Cyber–Physical–Social Systems, ITSs are highly integrated into organisations that deliver critical services to society. Subsequently, emergent behaviour, such as in the form of an outage, can cause cascading failure not only in the cyber domain, but into the physical and social domains as well. Subsequently, building C-CPSSs and ITSs that are resilient to such phenomena and can have their missions assured is a top priority for critical service sectors.

There are limited existing synthetic datasets within the literature that can be used to test and verify cascading-failure dynamics within the context of C-CPSSs. This research offers a new ITS dataset, ODIN-DAWN, that was generated from established architecture standards and training scenario databases. The research value of this new dataset comes from its realistic and relevance to real-world C-CPSS applications, where nodal criticality, cascading failure, and mission assurance functions must all be considered as part of service delivery. This dataset has the opportunity to grow and expand beyond its current form. Whilst only three service packages from the ARC-IT architecture were applied, there is the potential for service extension to best meet the experimentation needs of a variety of different stakeholders across government, commercial, and research sectors. Examples include international border management, ITS data warehousing, roadway construction, and emissions management [3].

This research presented a new programmatic model that can be used to simulate the cascading-failure effects of nodes within a C-CPSS and demonstrated its effectiveness with two intelligent transport system use cases. The first applied the ARC-IT architecture to demonstrate the critical interdependencies between transport services across generalised ITS services, and the second applied specific use cases from ARC-IT to the U.S. Army’s ODIN scenario environment to simulate real-world cascading-failure applications. We developed a dataset that applied the ARC-IT architecture to ODIN’s information environment, called ODIN-DAWN, forming a realistic ITS use-case scenario that can be employed in future research.

This new model expanded the current understanding of C-CPSS research through the identification of critical nodes through a combination of graph theory and an analysis of cascading failure percolation across the network. This combination provided a greater understanding than traditional graph theory alone. Additionally, the model’s novel approach of including social nodes as part of a C-CPSS instead of traditional cyber–physical system approaches increased the accuracy of simulation effects by up to 30%, highlighting the immense importance of considering social factors within these systems. This represents significant research value for future work, where social factors for simulations need to be accurately measured and included within the C-CPSS.

There is a future opportunity to integrate this C-CPSS cascading-failure simulation model into a tabletop or simulated exercise in order to validate its accuracy and increase its real-world value to training scenarios applied to target stakeholders. Noting that the ODIN data are sourced from the U.S. Army, integrating the new ODIN-DAWN dataset with the C-CPSS cascading-failure programmatic model would give the opportunity to test business continuity plans such as PACE (Primary, Alternate, Contingency, and Emergency) communications plans, in addition to branch plans, when outages cause critical service degradation but mission priorities must still be achieved.

The model could also be applied to tabletops or simulated exercises outside of the military context and would be of particular value to organisations in critical service sectors such as banking, government, healthcare, and National Critical Infrastructure (NCI). Applying the programmatic model offers the opportunity for key stakeholders to consider the feasibility and validity of their existing disaster management plans and to exercise these plans against a cascading failure outage at each order of consequence. These activities provide valuable feedback to cyber-security managers about what nodes require hardening but also inform management and decision-makers about the highest-risk service sectors within their organisation that need to be protected and assured.

Ultimately, the model developed and the datasets simulated show the significant effect that emergent behaviours in ITS and C-CPSS can have to wider systems of systems. It clearly shows the impact that including social nodes in cascading-failure analysis has on improving the accuracy of consequence percolation calculations. It provides a method through which the U.S. ARC-IT standard can be applied to cascading failure scenarios to validate ITS architectures and offers the research community a working scenario as part of the ODIN environment to demonstrate its capability. As ITS implementations and research progress, there will be an increased need for datasets in this field.

This research propels forward the C-CPSS body of knowledge and highlights the pertinent need for resilient cyber-security design that meets mission assurance priorities.

## Figures and Tables

**Table 1:** ODIN-DAWN data excerpt.

Node Name	isCyber	isPhysical	isSocial	Architecture Layer
Khorathidin Transportation Information Center	1	1	0	Physical
CPI V01 EQ01—Commercial Vehicle OBE	1	1	0	Physical
Napat Srisuwan—Fleet-Freight Manager	0	1	1	Physical
BRLS0001—Commercial Vehicle OBE	1	1	0	Physical
BT FlatPack 01—Freight Equipment	0	1	0	Physical
Pnom Pehn Ambulance 01—Emergency Vehicle OBE	1	1	0	Physical
Inthira Saengsuwan—Emergency Personnel	0	1	1	Physical

**Table 2:** Python programmatic model functions.

Function	Description	Note
(1)	Given a node and a set order of consequences, calculate the impact of an outage to the system	ARC-IT enterprise, functional, and physical layers only.
(2)	Given a node and a set order of consequences, calculate the impact of an outage to the system whilst removing all social-only nodes	ARC-IT enterprise, functional, and physical layers only. Taking a cyber–physical system (CPS) approach instead of a CPSS approach.
(3)	Given a node, print out key data	ARC-IT enterprise, functional, and physical layers only.
(4)	Graph the output of the entire system	Visual output.
(5)	Sort cyber nodes by the highest reachability	ARC-IT physical layer only.
(6)	Sort cyber nodes by the biggest impact to reachability that social nodes in the system influence	ARC-IT physical layer only. Highlights social impact.
(7)	Sort cyber nodes by the highest degree	ARC-IT physical layer only.
(8)	Given a node and a set order of consequences, calculate the impact of an outage to the system	ARC-IT communication layer only. Is the layer equivalent to (1).
(9)	Given a node and a set order of consequences, calculate the impact of an outage to the system whilst removing all social-only nodes	ARC-IT communication layer only. The layer is equivalent to (2).
(10)	Sort Communication Profiles by highest instance of degree connectivity to links	ARC-IT communication layer only. The layer is equivalent to (7).

## Data Availability

The original data presented in the study are openly available in the ODIN-DAWN dataset at https://github.com/tmay22/ODIN-DAWN (accessed on 14 June 2025); Cascading Failure model at https://github.com/tmay22/C-CPSS-Cascading-Failure-ITS-Model (accessed on 14 June 2025); ARC-IT at https://www.arc-it.net/ (accessed on 3 March 2025); ODIN DATE World at https://odin.tradoc.army.mil/DATEWORLD (accessed on 7 May 2025).

## Abbreviations

The following abbreviations are used in this manuscript:

C-CPSS	Complex Cyber–Physical–Social–System
CPSS	Cyber–Physical–Social System
CPS	Cyber–Physical System
ITS	Intelligent Transport System
